# Endothelial IGF- 1R deficiency disrupts microvascular homeostasis, impairing skeletal muscle perfusion and endurance: implications for age-related sarcopenia

**DOI:** 10.1007/s11357-025-01653-2

**Published:** 2025-04-08

**Authors:** Adam Nyul-Toth, Santny Shanmugarama, Roland Patai, Rafal Gulej, Janet Faakye, Dorina Nagy, Mark Nagykaldi, Tamas Kiss, Tamas Csipo, Madison Milan, Shoba Ekambaram, Sharon Negri, Raghavendra Y. Nagaraja, Anna Csiszar, Jacob L. Brown, Holly Van Remmen, Anna Ungvari, Andriy Yabluchanskiy, Stefano Tarantini, Zoltan Ungvari

**Affiliations:** 1 Vascular Cognitive Impairment, Neurodegeneration and Healthy Brain Aging Program, Department of Neurosurgery, University of Oklahoma Health Sciences Center, Oklahoma City, OK USA; 2 Oklahoma Center for Geroscience and Healthy Brain Aging, University of Oklahoma Health Sciences Center, Oklahoma City, OK USA; 3 Doctoral College, Health Sciences Division, Semmelweis University, Budapest, Hungary; 4 International Training Program in Geroscience, Doctoral College, Health Sciences Division/Institute of Public Health and Preventive Medicine, Semmelweis University, Budapest, Hungary; 5 Institute of Translational Medicine, Semmelweis University, Budapest, Hungary; 6 HUN-REN-SU Cerebrovascular and Neurocognitive Diseases Research Group, Budapest, Hungary; 7 Pediatric Center, Semmelweis University, Budapest, Hungary; 8 Institute of Public Health and Preventive Medicine, Semmelweis University, Budapest, Hungary; 9 Aging & Metabolism Research Program, Oklahoma Medical Research Foundation, Oklahoma City, OK USA; 10 Oklahoma City VA Medical Center, Oklahoma City, OK USA; 11 Department of Biochemistry & Molecular Biology, University of Oklahoma Health Sciences Center, Oklahoma City, OK USA; 12 Department of Health Promotion Sciences, College of Public Health, University of Oklahoma Health Sciences Center, Oklahoma City, OK USA; 13 The Peggy and Charles Stephenson Cancer Center, University of Oklahoma Health Sciences Center, Oklahoma City, OK USA

**Keywords:** Aging, Insulin-like growth factor- 1, IGF- 1, IGF- 1R, Vascular function, Skeletal muscle, Microvasculature, Sarcopenia, Claudication, Endothelial dysfunction

## Abstract

Aging is associated with a progressive decline in circulating insulin-like growth factor- 1 (IGF- 1) levels in humans, which has been implicated in the pathogenesis of sarcopenia. IGF- 1 is an anabolic hormone that plays a dual role in maintaining skeletal muscle health, acting both directly on muscle fibers to promote growth and indirectly by supporting the vascular network that sustains muscle perfusion. However, the microvascular consequences of IGF- 1 deficiency in aging muscle remain poorly understood. To elucidate how impaired IGF- 1 input affects skeletal muscle vasculature, we examined the effects of endothelial-specific IGF- 1 receptor (IGF- 1R) deficiency using a mouse model of endothelial IGF- 1R knockdown (VE-Cadherin-CreER^T2^/Igf1r^f/f^ mice). These mice exhibited significantly reduced skeletal muscle endurance and attenuated hyperemic response to acetylcholine, an endothelium-dependent vasodilator. Additionally, they displayed microvascular rarefaction and impaired nitric oxide-dependent vasorelaxation, indicating a significant decline in microvascular health in skeletal muscle. These findings suggest that endothelial IGF- 1R signaling is critical for maintaining microvascular integrity, muscle perfusion, and function. Impaired IGF- 1 input to the microvascular endothelium may contribute to reduced muscle blood flow and exacerbate age-related sarcopenia. Enhancing vascular health by modulating IGF- 1 signaling could represent a potential therapeutic strategy to counteract age-related muscle decline.

## Introduction

Aging is accompanied by a progressive decline in skeletal muscle strength and mass, a condition known as sarcopenia [1]. Sarcopenia is a major global public health concern due to its association with frailty, loss of independence, increased risk of falls, and higher morbidity and mortality rates. As populations age, sarcopenia imposes a significant socioeconomic burden, contributing to rising healthcare costs and disability rates in industrialized countries [2]. Despite its clinical importance, effective therapeutic strategies for preventing or reversing sarcopenia remain limited, underscoring the need for a deeper understanding of its underlying mechanisms [2].

The development of sarcopenia is driven by both intrinsic and extrinsic cellular processes, including dysregulated protein synthesis and degradation, chronic inflammation, impaired blood flow [3–5], mitochondrial dysfunction, and hormonal changes [1]. Among the key anabolic regulators of muscle homeostasis, insulin-like growth factor- 1 (IGF- 1) has emerged as a critical factor influencing both muscle mass maintenance and vascular function [6–16].

IGF- 1, an anabolic hormone primarily produced by the liver, is well known for its pro-growth, vasoprotective, and anti-aging properties [17–24]. It directly promotes muscle protein synthesis, prevents atrophy, and enhances regenerative capacity while also exerting indirect effects by regulating vascular function [7, 25, 26]. Notably, IGF- 1 enhances endothelial nitric oxide (NO) production [17, 18, 22, 27–34], promotes angiogenesis and maintains microvascular density [21, 35–42], and supports functional hyperemia in multiple organs [18, 20, 22, 29, 43], which mechanisms are also essential for meeting the metabolic demands of active muscle. However, circulating IGF- 1 levels decline significantly with age, a process linked to anabolic resistance, impaired vascular function, and skeletal muscle dysfunction [18].

While the muscle-intrinsic effects of IGF- 1 have been extensively studied [16, 25, 26], its role in maintaining skeletal muscle microvascular function during aging remains poorly understood. Endothelial cells, which highly express the IGF- 1 receptor (IGF- 1R) [22, 40, 44], play a fundamental role in regulating blood flow and vascular homeostasis. Previous studies have demonstrated that IGF- 1 deficiency contributes to vascular rarefaction and impaired vasodilation in the brain [18, 21, 29, 44], yet the consequences of IGF- 1R deficiency in skeletal muscle endothelium remain unclear.

To address this gap, we investigated how endothelial-specific IGF- 1R signaling regulates skeletal muscle vascular function using a mouse model of endothelial IGF- 1R knockdown (*VE-Cadherin-Cre*^*ERT2*^*/Igf1r*^*f/f*^) [22, 31, 44]. We hypothesized that disrupting endothelial IGF- 1R signaling would impair functional hyperemia, reduce microvascular density, and compromise skeletal muscle endurance, replicating aspects of the aging phenotype. To test this, we conducted fatigue and endurance tests, assessed skeletal muscle strength ex vivo, measured endothelium-mediated blood flow in the hind limb using laser speckle contrast imaging, and analyzed skeletal muscle capillarization via immunofluorescent labeling.

Our findings provide novel insights into the microvascular consequences of age-related IGF- 1 deficiency and highlight endothelial IGF- 1R as a potential therapeutic target for preventing vascular dysfunction and sarcopenia in aging muscle.

## Methods

### Animal model and experimental design

To generate an endothelial-specific IGF- 1 receptor (IGF- 1R) knockdown model, we utilized *Igf1r*^*f/f*^ (B6;129-Igf1rtm2 Arge/J; *loxP* sites flanking exon 3) and *VE-Cadherin-Cre *^*ERT2*^ (B6.FVB-Tg(Cdh5-cre)7Mlia/J; Stock No: 006137) mice, both obtained from Jackson Laboratories. Mice were housed in specific pathogen-free (SPF) conditions (including helicobacter- and parvovirus-free status) in the Rodent Barrier Facility at the University of Oklahoma Health Sciences Center (OUHSC). They were maintained in Allentown XJ cages (3–4 per cage) with Anderson’s Enrich-o-cob bedding (Maumee, OH) under controlled temperature (21 °C) and lighting conditions (14-h light/10-h dark cycle for breeding; 12-h light/12-h dark cycle for post-weaning maintenance). Animals had ad libitum access to irradiated bacteria-free standard rodent chow (5053 Pico Lab, Purina Mills, Richmond, IN) and reverse osmosis-filtered water.

For breeding, male *VE-Cadherin-Cre*^*ERT2*^ mice were crossed with female *Igf1r*^*f/f*^ mice to generate *VE-Cadherin-Cre*^*ERT2*^*/Igf1r*^+*/−*^ males, which were subsequently bred with *Igf1r*^*f/f*^ females to establish the *VE-Cadherin-Cre*^*ERT2*^*/IGF1R* homozygous floxed founder colony, following our previously described protocol [17, 45]. These mice were then bred to generate experimental cohorts of 1) *VE-Cadherin-Cre*^*ERT2*^*/Igf1r*^*f/f*^ (VECAD x IGF- 1R KD) and 2) *Cre-*/*Igf1r*^*f/f*^ (WT) control mice. To induce endothelial-specific IGF- 1R knockdown, 3-month-old mice received intraperitoneal tamoxifen injections (75 mg/kg body weight, dissolved in corn oil) for 5 consecutive days. Experiments were conducted on middle-aged mice (13–18 months old) to model accelerated age-related vascular and skeletal muscle changes. All experimental procedures were approved by the Institutional Animal Care and Use Committee (IACUC) of the University of Oklahoma Health Sciences Center (OUHSC) and conducted in compliance with NIH guidelines for the care and use of laboratory animals.

### Behavioral tests

#### Treadmill graded exercise test

**Fig. 1:**
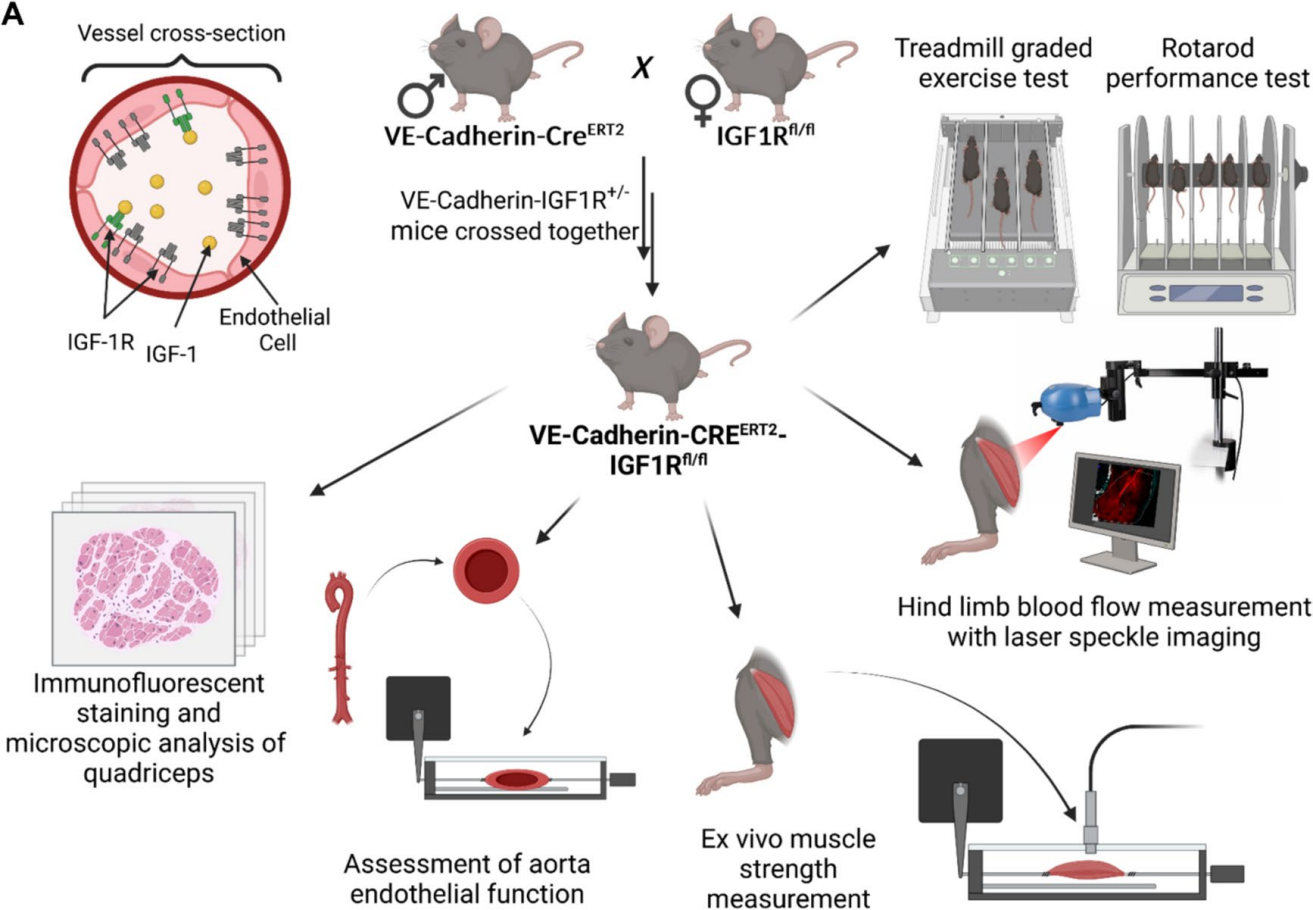
Breeding strategy and experimental methods for assessing the role of endothelial IGF- 1R in skeletal muscle function. Schematic representation of the breeding strategy used to generate endothelial-specific IGF- 1R knockout (VE-Cadherin-CreERT2/Igf1rf/f) mice. VE-Cadherin-CreERT2 mice were crossed with Igf1rf/f mice to obtain experimental cohorts for functional analysis. The inset (left) illustrates vascular endothelial IGF- 1R signaling, highlighting IGF- 1 and IGF- 1R localization in endothelial cells. Shown is also the experimental workflow for assessing the impact of endothelial IGF- 1R deficiency on skeletal muscle function. Motor coordination and fatigue resistance were evaluated using the rotarod performance test and treadmill graded exercise test. Skeletal muscle blood flow was assessed via hind limb laser speckle imaging, while quadriceps vascularization and muscle fiber structure were analyzed by immunofluorescent staining and confocal microscopy. Endothelial function was further investigated by measuring aortic vasorelaxation in isolated vessel preparations. In vitro skeletal muscle strength measurements were also performed

To assess exercise capacity and endurance, WT and VECAD × IGF- 1R KD mice were tested using a LE8700 TS Treadmill (Panlab Harvard Apparatus; Fig. 1). Following body weight measurement, mice were placed in individual running slots, and a graded running protocol was initiated. The protocol started with a 0% incline at 7.5 cm/s for 10 min, followed by a 5% incline at 12 cm/s for 30 min, then a 5% incline at 16.5 cm/s for another 30 min. After this phase, the speed was increased by 4.5% every 30 min until reaching 34.5 cm/s.

Each trial continued until the mouse was unable to sustain running for more than 10–15 s, despite gentle encouragement. Key performance metrics—including total running time, distance covered, and finishing speed—were recorded for each animal. Additionally, exercise work (J) and power (W) were calculated based on individual body weight using the equations:

$$\text{Work}=\text{Force}\times \text{Total Distance}$$

$$\text{Power }(W)=\frac{\text{Work}}{\text{Total Time}}$$

#### Rotarod performance test

To evaluate motor coordination, balance, and skill learning, mice underwent a four-lane automated rotarod test (Fig. 1) following previously established protocols [46, 47]. On the day of testing, mice were acclimated to the testing room for at least 15 min before trials began and were maintained in their home cages between trials to minimize stress.

Motor coordination was assessed using a Rotamex rotarod (Columbus Instruments, Columbus, OH) with an acceleration-based protocol. During the pre-training phase, mice were placed on the moving rotarod at 10 rpm and remained for 3 min to familiarize them with the apparatus. The test phase consisted of three trials per day with 15-min intervals over four consecutive days. The rotarod started at 4 rpm and gradually accelerated to 40 rpm over 300 s.

Latency to fall (measured in seconds) was recorded using an infrared beam sensor, along with the maximum rpm sustained by each mouse. Final performance outcomes were analyzed based on the results from day 4.

### Grip strength measurement

The grip strength test was used to assess maximal forelimb muscle strength in mice. Measurements were performed using a Chatillon Ametek Force Measurement grip strength meter (Brooklyn, NY), following the manufacturer’s recommendations. Each mouse underwent three consecutive trials, conducted by the same investigator to ensure consistency. The highest recorded grip strength value was used for subsequent analysis.

### Hind limb blood flow measurement with laser speckle imaging

To assess endothelium-dependent vasodilation in the quadriceps muscle, five animals per group (WT and VECAD × IGF- 1R KD) were anesthetized using isoflurane (induction 3–4%, maintenance 1.5–2%) in oxygen and mechanically ventilated to ensure stable respiration. The quadriceps muscle was surgically exposed and positioned under a laser speckle contrast imager for real-time perfusion assessment.

Following a 5-min baseline recording, the muscle was superfused with a 10⁻^5^ M acetylcholine (ACh) solution to induce endothelium-mediated vasodilation, and perfusion was recorded for an additional 5 min post-superfusion. Each animal underwent three independent trials, and recorded data were analyzed to determine the average response, maximal response, and area under the curve (AUC) to quantify changes in muscle blood flow dynamics.

### Ex vivo skeletal muscle strength measurement

To evaluate intrinsic muscle contractile properties, ex vivo force measurements were performed on isolated extensor digitorum longus skeletal muscle using standardized protocols [48]. The extensor digitorum longus muscles were carefully dissected from WT and VECAD × IGF- 1R KD mice while continuously superfused with oxygenated Krebs–Henseleit buffer (95% O₂, 5% CO₂, pH 7.4, 37 °C) to maintain physiological conditions. Muscles were mounted in a dual-mode muscle lever system (Aurora Scientific, ON, Canada), with one end attached to a fixed post and the other connected to a force transducer. After an initial equilibration period, optimal muscle length (*L*₀) was determined by progressively adjusting tension until maximal twitch force was achieved. Isometric force production was assessed by delivering supramaximal electrical stimulation (0.5 ms pulse width, 120 Hz, 300 ms train duration) via platinum electrodes. Specific force (N/cm^2^) was calculated by normalizing absolute force (N) to cross-sectional area (CSA, cm^2^). Muscle function was further assessed through measurements of maximum tetanic force and specific force allowing for direct comparison between experimental groups.

### Assessment of aorta endothelial function

Endothelium-dependent vasorelaxation was assessed in isolated aortic ring preparations from perfused WT and VECAD × IGF- 1R KD animals to evaluate the impact of endothelium-specific IGF- 1 signaling deficiency on vascular function. Aortic vasorelaxation was measured as previously described [49].

In brief, aortas were excised, cleaned of surrounding connective tissue, and cut into 1.5-mm ring segments, which were then mounted in myograph chambers (Danish Myo Technology A/S, Inc., Denmark) for isometric tension measurement. The vessels were continuously superfused with Krebs buffer solution (118 mM NaCl, 4.7 mM KCl, 1.5 mM CaCl₂, 25 mM NaHCO₃, 1.1 mM MgSO₄, 1.2 mM KH₂PO₄, and 5.6 mM glucose) at 37 °C, gassed with 95% air and 5% CO₂.

After a 1-h equilibration period, during which optimal passive tension was applied (determined from the vascular length-tension relationship), aortic rings were pre-contracted with 10⁻⁶ M phenylephrine until a stable plateau phase was reached. Endothelium-dependent relaxation was then evaluated by cumulative administration of acetylcholine (ACh) at concentrations ranging from 10⁻⁹ to 10⁻^5^ M, and the resulting changes in tension were recorded.

### Immunofluorescent staining and microscopic analysis of quadriceps

To analyze skeletal muscle microvasculature and fiber structure, animals were perfused, and the hind limb quadriceps muscle was fixed in 4% ice-cold paraformaldehyde (PFA). Tissues were then embedded in OCT compound, and 35-μm sagittal sections were obtained using a cryostat and stored at − 20 °C on microscopy slides. Before immunostaining, sections were rinsed with Tris-buffered saline (TBS) and permeabilized with 0.05% Tween- 20 in TBS. Antigen retrieval was performed by incubating the sections in 10 mM citrate buffer (10 mM sodium citrate, 0.05% Tween- 20, pH 6.0) at 90 °C for 20 min, followed by three washes with TBS-Tween (TBST). To reduce non-specific binding, sections were blocked for 2 h at room temperature in a solution containing 5% bovine serum albumin (BSA) and 1% fish gelatin in TBS.

For vascular and muscle fiber visualization, sections were incubated overnight at 4 °C with primary antibodies: rat monoclonal anti-mouse endomucin (1:50, Invitrogen) to label endothelial cells and anti-mouse laminin (1:50, Invitrogen) to delineate muscle fibers. Following three 5-min washes with TBST, sections were incubated for 2 h at room temperature with fluorophore-conjugated secondary antibodies: goat anti-rat IgG Alexa Fluor 488 (Invitrogen) and goat anti-rabbit IgG Alexa Fluor 532 (Invitrogen). Sections were washed three times with TBST and counterstained with DAPI (5 mg/mL, 1:10,000, Invitrogen) for 5 min to label nuclei.

After a final wash, sections were mounted using ProLong GOLD antifade mounting medium (Invitrogen, Thermo Fisher Scientific). Imaging was performed using a Leica SP8 MP confocal laser scanning microscope with uniform settings and laser power to ensure consistency across samples. The number of blood vessels and laminin-positive muscle fibers per region of interest (ROI) was quantified using adaptive thresholding and manual counting.

### Statistics

Statistical analyses were performed using GraphPad Prism 8.0.1 (Dotmatics). Depending on the experiment, data were analyzed using either an unpaired or paired t-test, or a two-way ANOVA followed by a Fisher’s LSD post hoc test when multiple comparisons was required.

Differences were considered statistically significant at *p* < 0.05. Data are presented as mean ± standard error of the mean (SEM) unless otherwise specified. In cases where individual data points are displayed, results are shown as bar graphs, dot plots, or box plots with interquartile distributions. The number of biological and technical replicates for each experiment is indicated in the figure legends.

## Results

### Rotarod performance and fatigue resistance

**Fig. 2:**
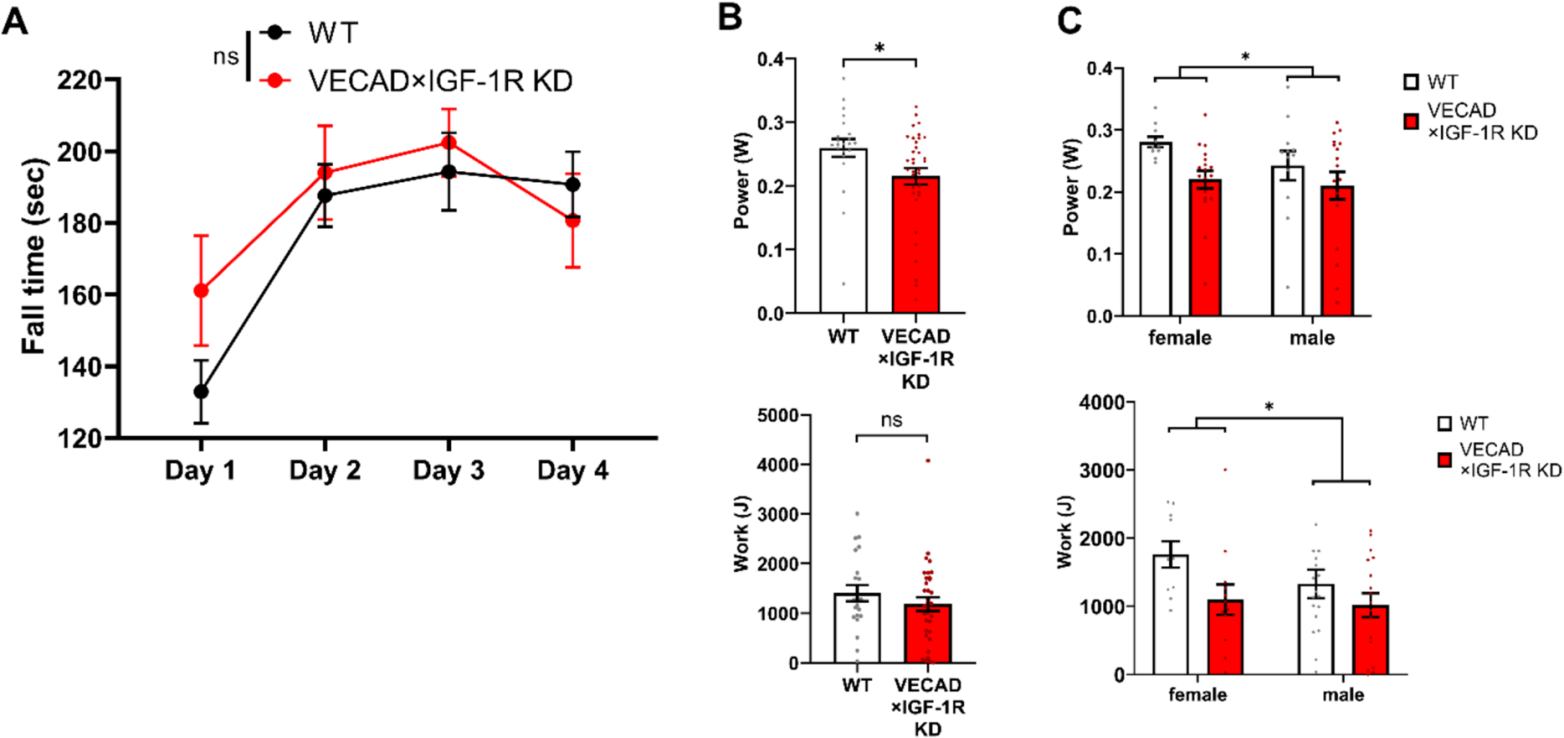
Endothelial IGF- 1R deficiency impairs muscle endurance and fatigue resistance without affecting motor coordination. **A** Motor coordination and balance were assessed using a rotarod test over four consecutive days. No significant differences in fall time were observed between WT (black) and VECAD × IGF- 1R KD (red) mice, indicating that endothelial IGF- 1R deficiency does not impair motor coordination or balance (*n* = 22 WT, *n* = 35 KD; two-way ANOVA). **B** Treadmill fatigue resistance test revealed a significant reduction in muscle power in VECAD × IGF- 1R KD mice compared to WT controls (*p* < 0.05, upper panel). A trend toward reduced total work performed was observed in KD mice but did not reach statistical significance (*p* = ns, lower panel; unpaired Student’s t-test). **C** Sex-specific analysis of treadmill performance. Two-way ANOVA revealed a significant effect of IGF- 1R KD on muscle power (*p* < 0.05), with no significant sex effect (upper panel). However, total work performed was significantly reduced in female KD mice compared to their WT counterparts (*p* < 0.05, lower panel), suggesting a greater susceptibility of female mice to IGF- 1R deficiency-induced fatigue resistance impairment. Data are presented as mean ± SEM. *p* < 0.05 was considered statistically significant

To assess motor coordination and balance, VECAD × IGF- 1R KD and WT mice were subjected to the rotarod test over four consecutive days. There were no significant differences in fall time between groups, indicating that endothelial IGF- 1R deficiency does not impair motor coordination or balance (Fig. 2A). Both WT and KD mice exhibited similar learning curves, with fall time increasing progressively from day 1 to day 4.

To evaluate muscle fatigue resistance and endurance capacity, treadmill performance was assessed by measuring muscle power (W) and total work performed (J). VECAD × IGF- 1R KD mice displayed significantly lower muscle power compared to WT controls (*p* < 0.05, Fig. 2B, upper panel). While a trend toward reduced total work was observed in KD mice, the difference did not reach statistical significance (Fig. 2B, lower panel).

Given potential sex-related differences in fatigue resistance and the muscle [16, 50] and anti-aging effects of IGF- 1 [24, 51], muscle power and work were analyzed separately in male and female mice. Two-way ANOVA revealed a significant effect of IGF- 1R KD on muscle power (*p* < 0.05) but no significant effect of sex (Fig. 2C, upper panel). However, the reduction in muscle work was more pronounced in female KD mice compared to males (*p* < 0.05, Fig. 2C, lower panel), suggesting a potential sex-specific vulnerability to endothelial IGF- 1R deficiency-induced muscle fatigue. These findings indicate that loss of endothelial IGF- 1R signaling impairs muscle endurance and fatigue resistance without affecting motor coordination or balance, with females exhibiting a greater susceptibility to functional decline.

### Endothelial IGF- 1R knockdown impairs skeletal muscle strength without causing significant muscle atrophy

**Fig. 3:**
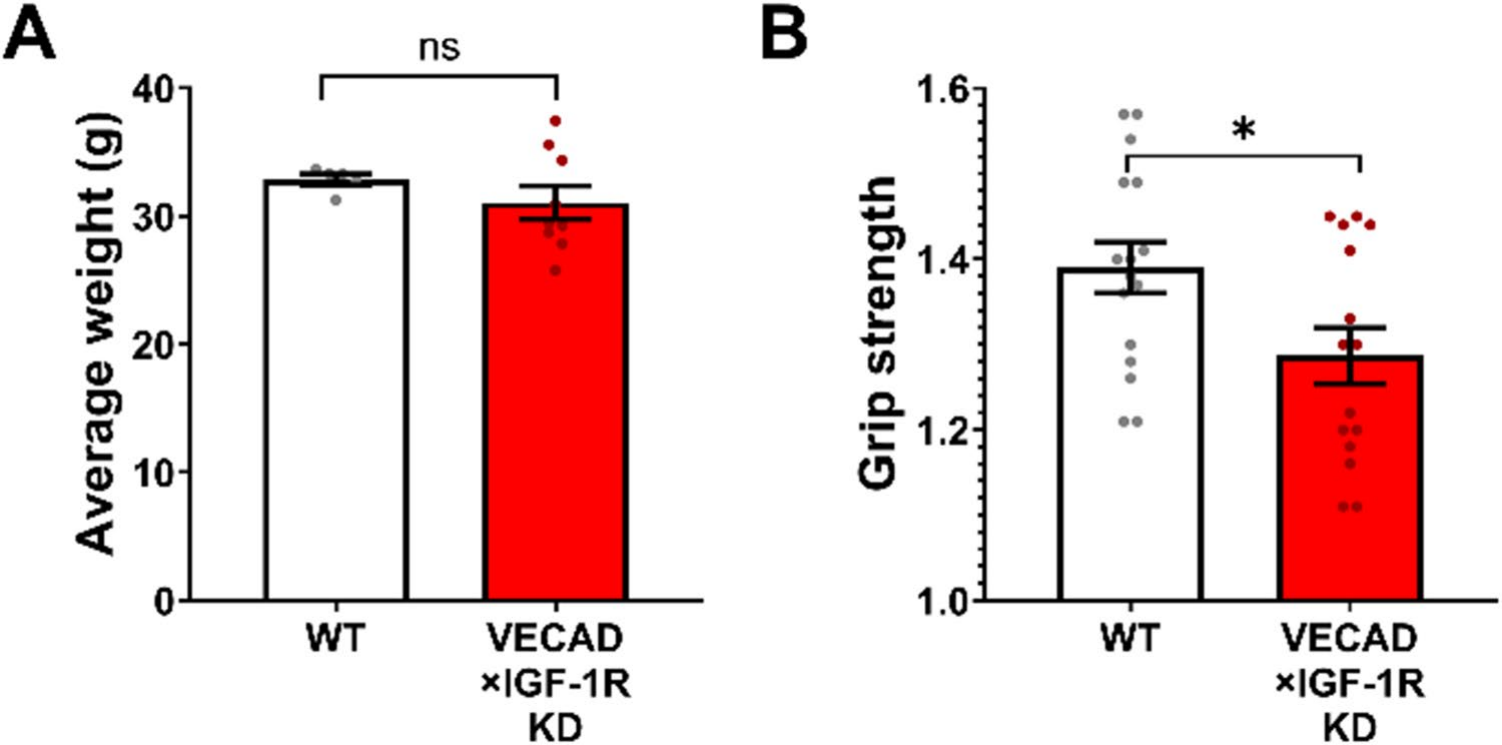
Endothelial-specific IGF- 1R knockdown impairs skeletal muscle strength without causing significant muscle atrophy. **A** Average body weight of WT (white) and VECAD × IGF- 1R KD (red) mice. No significant differences were observed between groups, indicating that endothelial IGF- 1R deficiency does not induce overt muscular atrophy (WT: *n* = 5, KD: *n* = 9; unpaired two-tailed t-test). **B** Grip strength test results show a significant reduction in the maximal forelimb strength of VECAD × IGF- 1R KD mice compared to WT controls (*p* < 0.05), suggesting that endothelial IGF- 1R signaling plays a role in maintaining muscle force generation (WT: *n* = 16, KD: *n* = 15; unpaired two-tailed t-test). Data are presented as mean ± SEM. *p* < 0.05 was considered statistically significant

Skeletal muscle mass plays a critical role in determining the maximal force output, and its reduction can severely impair muscle strength [52–55]. To determine whether endothelial IGF- 1R deficiency affects muscle mass, we compared the body weight of VECAD × IGF- 1R KD and WT mice. There were no significant differences between groups (Fig. 3A), suggesting that the loss of endothelial IGF- 1R does not induce overt muscular atrophy. However, grip strength testing revealed a significant reduction in maximal forelimb strength in VECAD × IGF- 1R KD mice compared to WT controls (*p* < 0.05, Fig. 3B). This decline in grip strength occurred independently of body weight, indicating that endothelial IGF- 1R signaling plays a critical role in maintaining muscle force generation, potentially through vascular contributions to muscle function rather than direct muscle atrophy.

### In vitromuscle strength is preserved in endothelial IGF- 1R knockdown mice

To determine whether the observed reduction in muscle strength was due to intrinsic muscle dysfunction or impaired vascular support, we assessed in vitro muscle contractile properties using isolated muscle preparations. Maximum tetanic force and specific force were measured in the extensor digitorum longus muscles from WT and VECAD × IGF- 1R KD mice.

**Fig. 4:**
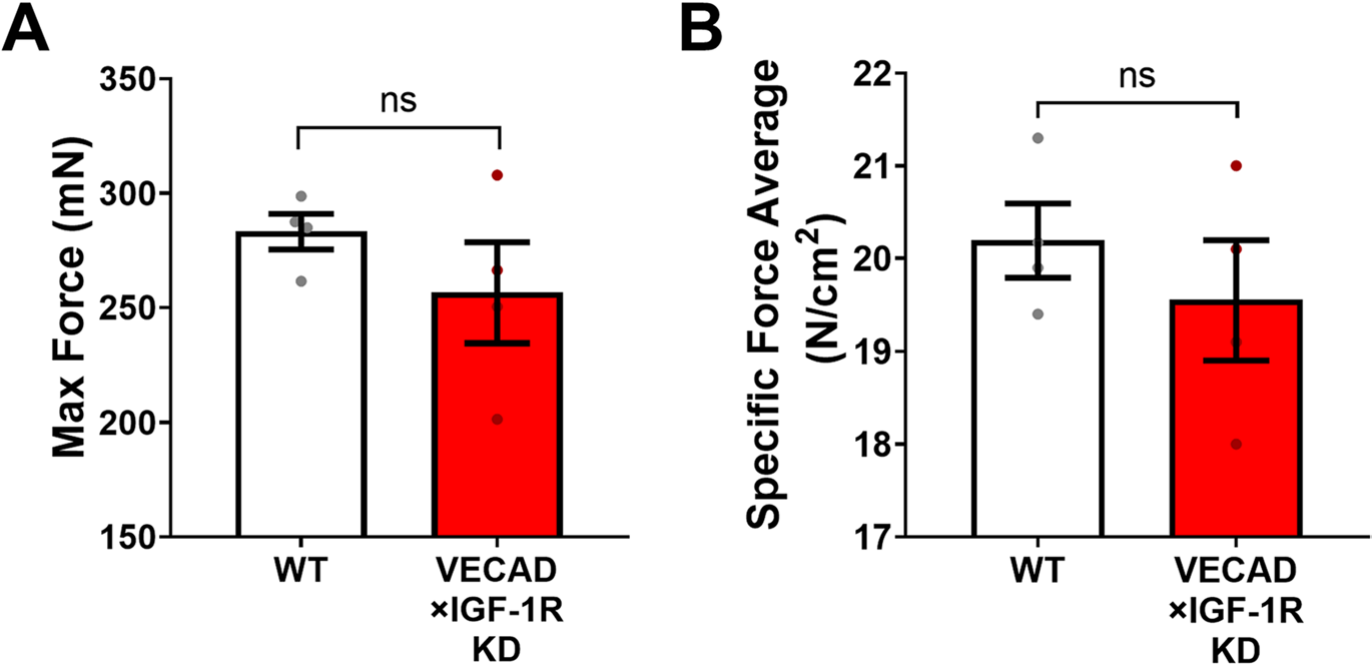
Endothelial IGF- 1R knockdown does not affect intrinsic muscle contractile strength. **A, B** Ex vivo muscle contractility assessment in isolated extensor digitorum longus muscles from WT (white) and VECAD × IGF- 1R KD (red) mice. **A** Maximum tetanic force and **B** specific force were measured using a muscle contractility assay to evaluate intrinsic muscle strength. No significant differences were observed between groups, indicating that endothelial IGF- 1R deficiency does not impair the intrinsic contractile properties of the skeletal muscle. These findings suggest that the observed reductions in endurance and muscle function in IGF- 1R KD mice are primarily due to vascular impairments rather than direct muscle weakness. Data are presented as mean ± SD. Statistical significance was set at *p* < 0.05 (unpaired two-tailed t-test)

There were no significant differences in maximum force production or specific force between groups, indicating that intrinsic muscle contractility was unaffected by IGF- 1R knockdown (Fig. 4). These findings suggest that the muscle weakness observed in vivo is not due to intrinsic deficits in muscle strength but rather may be due to external factors including impairments in vascular support and perfusion. These results support the hypothesis that endothelial IGF- 1R may play a critical role in maintaining functional muscle output via its effects on the microvasculature.

### Endothelial IGF- 1R knockdown impairs endothelium-dependent increases in skeletal muscle blood flow

**Fig. 5:**
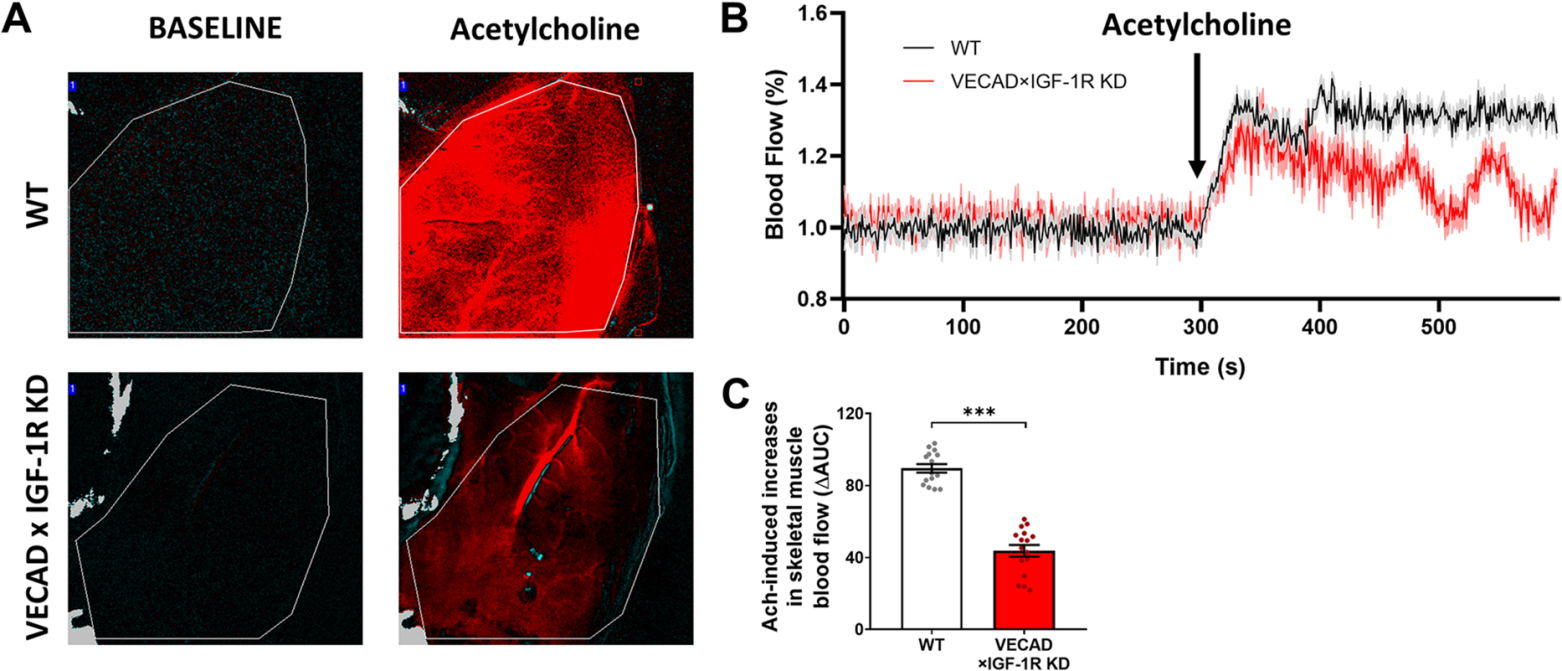
Endothelial IGF- 1R knockdown impairs acetylcholine-induced hyperemia in skeletal muscle. **A** Representative laser speckle contrast images of the gastrocnemius muscle before (baseline) and after acetylcholine (ACh) superfusion (10⁻^5^ M) in WT (top) and VECAD × IGF- 1R KD (bottom) mice. WT mice exhibited a strong vasodilatory response, whereas KD mice displayed a blunted hyperemic response, indicating impaired endothelial function. **B** Time-course of perfusion changes in response to ACh superfusion in WT (black) and KD (red) mice. VECAD × IGF- 1R KD mice showed attenuated blood flow increases compared to WT controls. **C** Bar graph quantifying the total vasodilatory response (area under the curve, AUC). VECAD × IGF- 1R KD mice exhibited a significantly lower AUC, suggesting an inadequate vascular response to ACh stimulation (*p* < 0.001). Data are presented as mean ± SEM. ***p < 0.001 (unpaired two-tailed t-test)

To determine whether endothelial IGF- 1R deficiency affects skeletal muscle microvascular function, we measured quadriceps muscle blood flow responses to superfusion of acetylcholine, an endothelium-dependent vasodilator, using laser speckle contrast imaging. At baseline, resting perfusion levels were comparable between WT and KD mice (Fig. 5A, left panels). However, following ACh superfusion (10⁻^5^ M), WT mice exhibited a robust increase in blood flow, whereas KD mice displayed a significantly blunted vasodilatory response (Fig. 5A, right panels). Time-course analysis confirmed that ACh-induced hyperemia was attenuated in KD mice compared to WT controls (Fig. 5B).

Vasodilatory responses to acetylcholine in the skeletal muscle vasculature biphasic, with the initial phase primarily mediated by endothelium-derived hyperpolarizing factors (EDHFs), while the plateau phase reflects endothelium-dependent nitric oxide (NO)-mediated vasodilation [56, 57]. Thus, to quantify the overall vasodilatory response, we assessed total perfusion changes over time using area under the curve (AUC) analysis. VECAD × IGF- 1R KD mice exhibited a significantly reduced AUC compared to WT controls (*p* < 0.001, Fig. 5E), indicating impaired functional hyperemia in skeletal muscle. These findings suggest that endothelial IGF- 1R signaling is essential for regulating skeletal muscle perfusion, and its loss results in diminished vasodilatory capacity, potentially contributing to reduced exercise performance and muscle endurance.

### Endothelial IGF- 1R knockdown impairs aortic endothelium-dependent vasorelaxation

**Fig. 6:**
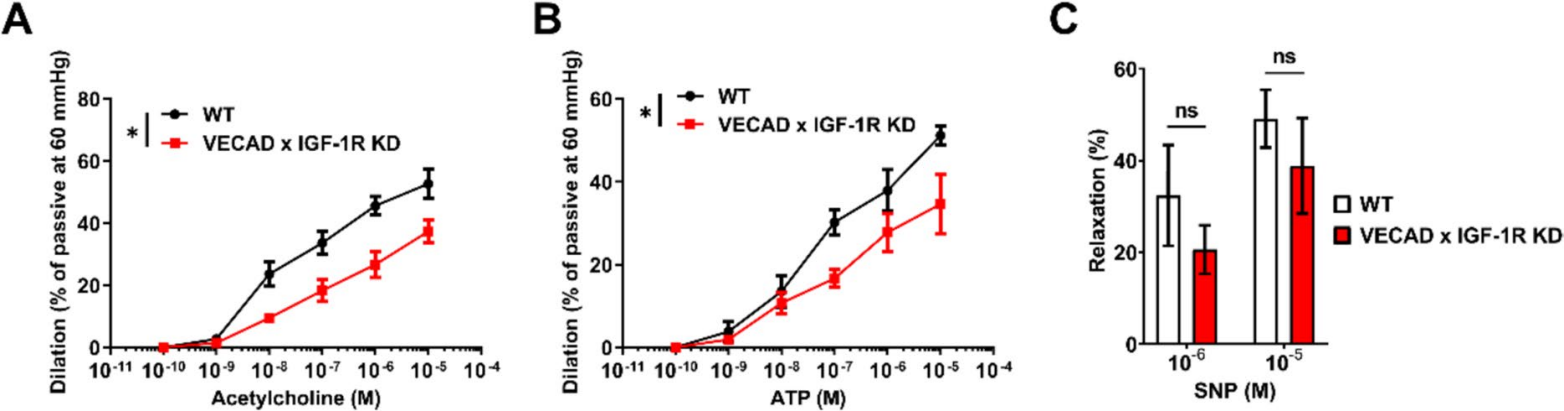
Endothelial IGF- 1R knockdown impairs endothelium-dependent vasorelaxation. **A–C** Vasorelaxation responses to acetylcholine (**A**), ATP (**B**), and sodium nitroprusside (SNP, **C**) were measured in isolated aortic ring preparations from WT (black) and VECAD × IGF- 1R KD (red) mice following phenylephrine precontraction (10⁻⁶ M). **A**, **B** ACh- and ATP-induced vasodilation were significantly reduced in VECAD × IGF- 1R KD mice compared to WT controls (*p* < 0.05), indicating endothelial dysfunction. **C** SNP-induced vasodilation was not significantly different between groups, suggesting that vascular smooth muscle responsiveness to NO remained intact. Data are presented as mean ± SD (WT: *n* = 4, KD: *n* = 4). *p* < 0.05 was considered statistically significant (paired t-test)

To further validate the impaired vascular endothelial function observed in skeletal muscle microcirculation, we assessed endothelium-dependent vasorelaxation in isolated aortic rings from WT and VECAD × IGF- 1R KD mice. Vasodilatory responses to acetylcholine (ACh), adenosine triphosphate (ATP), and responses to the NO donor sodium nitroprusside (SNP) were measured following phenylephrine-induced precontraction (10⁻⁶ M). ACh administration resulted in a significant reduction in endothelium-dependent relaxation in aortic rings from KD mice compared to WT controls (*p* < 0.05, Fig. 6A), indicating impaired nitric oxide (NO)-mediated vasorelaxation. Similarly, ATP-induced relaxation, another marker of endothelial function, was also significantly blunted in KD mice (*p* < 0.05, Fig. 6B). To determine whether this impairment extended to endothelium-independent mechanisms, we evaluated vasorelaxation responses to SNP, a direct NO donor. There were no significant differences in SNP-induced relaxation between WT and KD groups (Fig. 6C), suggesting that smooth muscle responsiveness to NO remained intact and that the observed vascular dysfunction was specifically endothelium-driven. These findings confirm that loss of endothelial IGF- 1R signaling impairs macrovascular function, consistent with the microvascular dysfunction observed in skeletal muscle circulation. This supports the hypothesis that endothelial dysfunction contributes to reduced muscle perfusion and impaired exercise performance in IGF- 1R-deficient mice.

### Endothelial IGF- 1R knockdown induces microvascular rarefaction in skeletal muscle

**Fig. 7:**
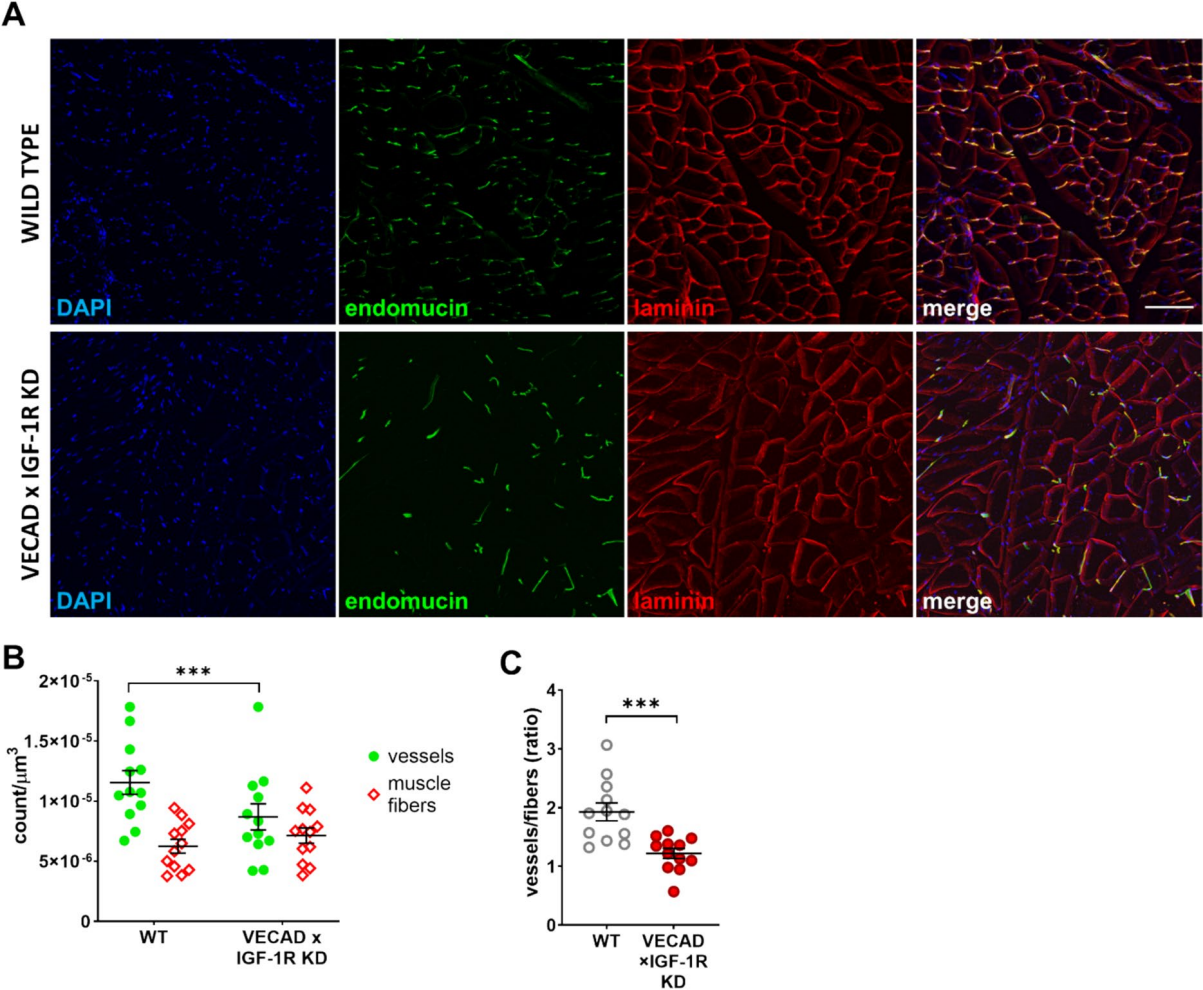
Endothelial IGF- 1R knockdown induces microvascular rarefaction in mouse quadriceps. **A** Representative immunofluorescence images of quadriceps muscle sections from WT (top) and VECAD × IGF- 1R KD (bottom) mice. Endothelial cells were labeled with endomucin (green), muscle fibers with laminin (red), and nuclei with DAPI (blue). VECAD × IGF- 1R KD mice exhibited a striking reduction in endomucin-positive vessels, indicating microvascular rarefaction. **B** Quantification of vascular and muscle fiber density in quadriceps muscle of WT and KD mice (*n* = 12 per group). Vascular density was significantly reduced in KD mice, whereas muscle fiber density remained unchanged. **C** Vessel-to-fiber ratio in quadriceps muscle. KD mice showed a robust decrease in vascularization relative to muscle fiber content, suggesting capillary rarefaction independent of muscle loss (*p* < 0.001). Data are presented as mean ± SEM. *p* < 0.05 was considered statistically significant

Since body weight and muscle mass remained unchanged in VECAD × IGF- 1R KD mice (Fig. 3B), we hypothesized that the reduced blood flow observed in these animals could result from vascular dysfunction and microvascular rarefaction. Given that adequate skeletal muscle vascularization is essential for maintaining sustained strength and endurance, we assessed microvascular density in the quadriceps using endomucin immunofluorescence staining. Quadriceps sections from KD mice exhibited markedly fewer endomucin-positive vessels compared to WT controls (Fig. 7A, B), indicating significant microvascular rarefaction. To evaluate whether this reduction in microvascular density was associated with muscle fiber loss, we performed laminin staining, which delineates individual muscle fibers. While muscle fiber density remained unchanged, the vessel-to-fiber ratio was significantly reduced in KD mice (*p* < 0.001, Fig. 7C), confirming that microvascular rarefaction occurred independently of muscle atrophy. These findings suggest that endothelial IGF- 1R signaling is essential for maintaining skeletal muscle capillary density, and its deficiency leads to impaired vascular supply, potentially contributing to exercise intolerance and muscle fatigue in KD mice.

## Discussion

Our study provides compelling evidence that endothelial IGF- 1R signaling plays a critical role in maintaining skeletal muscle vascularization, perfusion, and function. By selectively disrupting IGF- 1R in endothelial cells, we observed microvascular rarefaction, impaired functional hyperemia, and reduced muscle endurance, all occurring independently of muscle mass loss. These findings suggest that vascular health is essential for sustaining muscle function and that IGF- 1 deficiency may contribute to the early vascular component of age-related sarcopenia. While IGF- 1’s direct anabolic effects on muscle have been well established, our results highlight its underappreciated role in maintaining microvascular integrity and regulating muscle perfusion.

A key observation in our study was the significant reduction in neurotransmitter-mediated, endothelium-dependent hyperemia in IGF- 1R-deficient mice. This suggests that endothelial dysfunction limits the ability of skeletal muscle to regulate blood flow during activity, which may impair oxygen and nutrient delivery under increased metabolic demand [58]. Consistent with these findings, our present and previous studies confirmed that endothelium-dependent vasodilation is significantly impaired in IGF- 1R KD mice [22, 44]. These results reinforce the idea that IGF- 1 signaling maintains microvascular endothelial function through NO-dependent vasodilation [18]. Given that microvascular dysfunction is a hallmark of cardiovascular aging [59–61], our findings position endothelial IGF- 1 signaling as a potential target for preserving peripheral vascular health and muscle performance.

Beyond endothelial dysfunction, our study demonstrated significant capillary rarefaction in skeletal muscle of IGF- 1R KD mice. Importantly, muscle fiber density remained unchanged, confirming that the loss of vascular density occurred independently of muscle atrophy. This microvascular rarefaction is likely a key contributor to the reduced muscle endurance observed in IGF- 1R KD mice, as capillary networks are critical for sustaining aerobic metabolism and preventing muscle fatigue [58, 62, 63]. Our findings align with previous reports indicating that IGF- 1 signaling supports angiogenesis and microvascular integrity [21, 35–42], and its age-related decline leads to capillary loss, impaired blood flow, and progressive frailty. Interestingly, our data also suggest that female mice may exhibit greater susceptibility to endothelial IGF- 1R deficiency-induced reductions in endurance. Although our study was not powered to specifically interrogate sex differences, previous work has demonstrated that estrogen modulates IGF- 1 signaling [64–66] and plays a protective role in endothelial function [67]. The differential regulation of cellular mechanisms involved in vascular aging between males and females may partly explain the observed variation [67]. These sex-specific vascular responses in aging deserve further investigation in future studies.

**Fig. 8:**
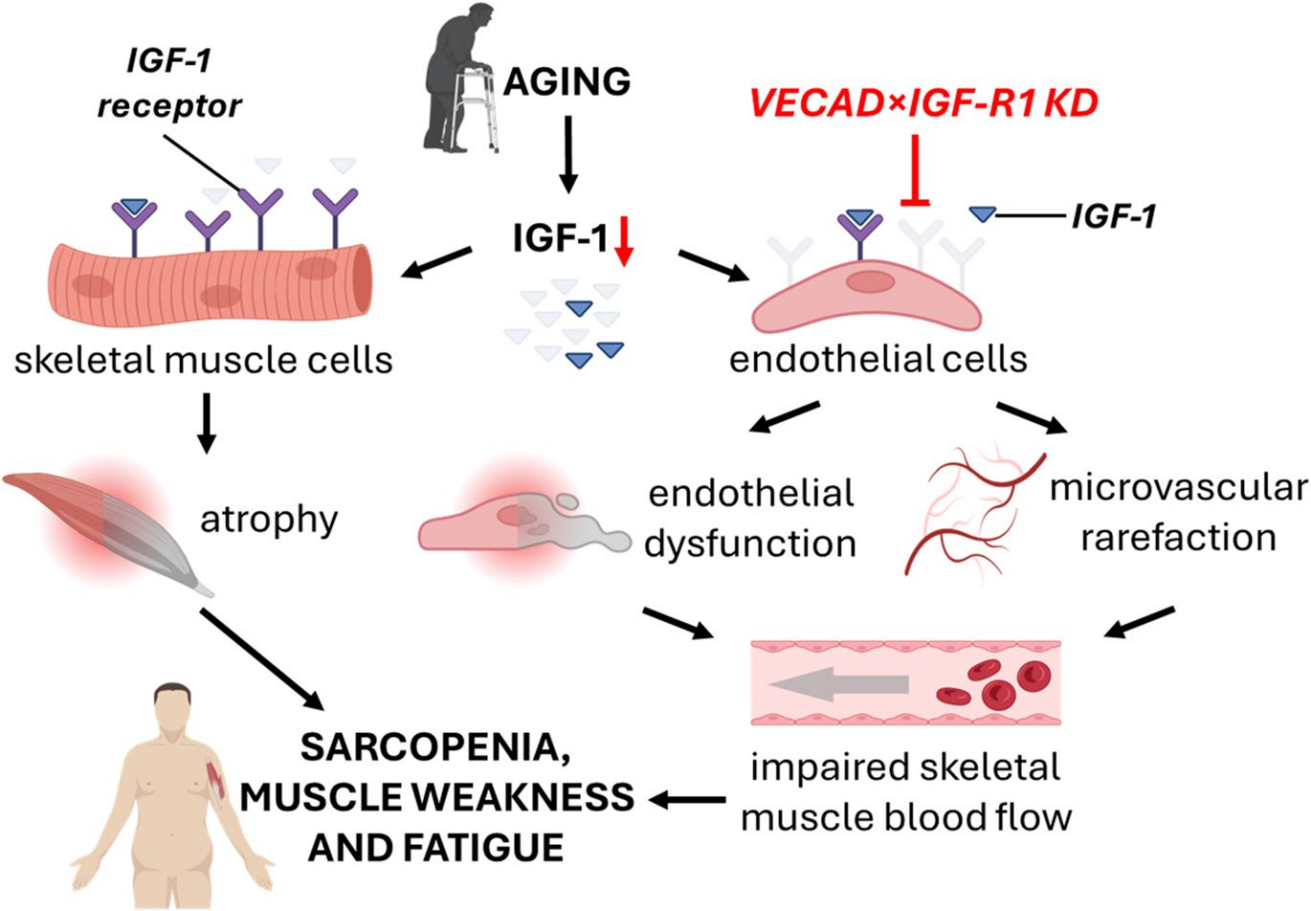
Schematic representation of how aging-related IGF- 1 deficiency contributes to sarcopenia, muscle weakness, and fatigue through direct muscle-intrinsic effects and vascular dysfunction. The direct pathway (left) illustrates that reduced IGF- 1 signaling in skeletal muscle leads to atrophy, which contributes to sarcopenia. The vascular pathway (right) demonstrates that impaired IGF- 1 input to endothelial cells promotes microvascular rarefaction and endothelial dysfunction, leading to impaired skeletal muscle blood flow, exacerbating muscle weakness and fatigue. The endothelial IGF- 1R knockout model (VECAD-IGF- 1R KO) mimics key aspects of age-related vascular decline. Figure was created with https://BioRender.com

Since circulating IGF- 1 levels naturally decline with age [18], our findings have important implications for age-related sarcopenia and microvascular aging (Fig. 8). The reduction in skeletal muscle capillarization observed in our IGF- 1R KD model mirrors the capillary rarefaction seen in aging muscle, which has been linked to exercise intolerance and muscle wasting in older adults [58]. This suggests that early microvascular impairments may precede overt muscle atrophy, potentially serving as a biomarker for sarcopenia risk.

Vascular aging has systemic consequences, affecting multiple organs by promoting oxidative stress, endothelial dysfunction, cellular senescence [44, 68, 69] and apoptosis [70], impaired angiogenesis [62, 71], morphological alterations of endothelial cells [72], and altered vasoregulatory mechanisms [73], ultimately leading to reduced perfusion and metabolic dysregulation [58, 74, 75]. IGF- 1 has long been recognized for its anabolic effects on skeletal muscle, but its vascular role has received less attention. Our findings highlight that vascular IGF- 1 signaling is essential for maintaining skeletal muscle homeostasis, extending beyond its role in muscle fiber hypertrophy. Given that IGF- 1R is highly expressed in endothelial cells [40], age-related IGF- 1 deficiency may directly contribute to microvascular dysfunction, reducing skeletal muscle perfusion and accelerating functional decline. This aligns with previous studies showing that IGF- 1 decline with age leads to endothelial dysfunction in multiple vascular beds, including the brain [18, 29], further supporting the notion that vascular IGF- 1 signaling is critical for overall tissue health.

Several mechanisms may underlie the vascular impairments observed in the absence of endothelial IGF- 1R signaling. IGF- 1 is known to exert anti-inflammatory and antioxidative effects on the vasculature [30, 76–78], and its deficiency has been linked to enhanced oxidative stress, endothelial senescence, and low-grade chronic inflammation—all of which are key features of vascular aging. Previous studies have shown that IGF- 1 deficiency can accelerate endothelial cell senescence [17] and increase susceptibility to inflammatory insults [76, 79], which may collectively contribute to the observed microvascular rarefaction and endothelial dysfunction in our model. Further mechanistic studies dissecting these downstream pathways would be highly informative.

While our endothelial IGF- 1R knockdown model offers a valuable tool to study the vascular contributions to sarcopenia, it does not fully recapitulate the complex interplay of systemic, metabolic, and hormonal factors involved in human aging. Additional limitations include the potential involvement of compensatory mechanisms, such as upregulation of alternative growth factors or inflammatory mediators, which may influence muscle function independently or synergistically. Nonetheless, the observed vascular phenotype in our model closely mirrors early age-related microvascular dysfunction seen in older adults, including reduced perfusion and endothelial impairment.

Our findings have important translational implications for aging and age-related muscle decline. Since IGF- 1R signaling is crucial for maintaining vascular health, interventions that enhance IGF- 1 bioavailability, improve endothelial function, or promote angiogenesis could help mitigate vascular contributions to sarcopenia. Aerobic exercise and dietary interventions have been shown to elevate endogenous IGF- 1 levels and support vascular health and may represent effective non-pharmacologic approaches to delay or mitigate vascular contributions to sarcopenia. Lifestyle interventions such as exercise [41, 80, 81] and dietary modifications [82, 83] may offer promising avenues for preserving muscle function in aging individuals. Further investigations are needed to elucidate the molecular mechanisms underlying IGF- 1R-mediated endothelial dysfunction, including oxidative stress [76–78], inflammation [30], pathways involved in cellular senescence [17], and metabolic alterations [79, 84, 85]. Additionally, the long-term consequences of endothelial IGF- 1R deficiency on skeletal muscle function should be explored, as chronic vascular insufficiency could eventually contribute to muscle wasting and frailty.

Taken together, our study highlights the essential role of endothelial IGF- 1R signaling in skeletal muscle vascular health and function. Deficient IGF- 1 input to vascular endothelial cells leads to impaired vasodilation, microvascular rarefaction, and reduced muscle endurance, without significant muscle atrophy. These findings provide new insights into the vascular contributions to sarcopenia and suggest that targeting IGF- 1 signaling in the endothelium may be a promising strategy to prevent age-related muscle decline. Importantly, the VECAD × IGF- 1R KD model recapitulates key features of early microvascular aging prior to the onset of overt muscle atrophy, making it a useful experimental system for studying pre-sarcopenia and identifying early vascular biomarkers of functional decline.

## Data Availability

N/A.
